# Field Balancing of Magnetically Levitated Rotors without Trial Weights

**DOI:** 10.3390/s131216000

**Published:** 2013-11-25

**Authors:** Jiancheng Fang, Yingguang Wang, Bangcheng Han, Shiqiang Zheng

**Affiliations:** 1 Key Laboratory of Fundamental Science for National Defense, Beihang University, Beijing 100191, China; E-Mail: fangjiancheng@buaa.edu.cn (J.F.); hanbangcheng@buaa.edu.cn (B.H.); zhengshiqiang@buaa.edu.cn (S.Z.); 2 Science and Technology on Inertial Laboratory, Beihang University, Beijing 100191, China; 3 School of Instrument Science and Opto-Electronics Engineering, Beihang University, Beijing 100191, China

**Keywords:** field balancing, magnetically levitated rotor, no trial weights, unbalance compensation control

## Abstract

Unbalance in magnetically levitated rotor (MLR) can cause undesirable synchronous vibrations and lead to the saturation of the magnetic actuator. Dynamic balancing is an important way to solve these problems. However, the traditional balancing methods, using rotor displacement to estimate a rotor's unbalance, requiring several trial-runs, are neither precise nor efficient. This paper presents a new balancing method for an MLR without trial weights. In this method, the rotor is forced to rotate around its geometric axis. The coil currents of magnetic bearing, rather than rotor displacement, are employed to calculate the correction masses. This method provides two benefits when the MLR's rotation axis coincides with the geometric axis: one is that unbalanced centrifugal force/torque equals the synchronous magnetic force/torque, and the other is that the magnetic force is proportional to the control current. These make calculation of the correction masses by measuring coil current with only a single start-up precise. An unbalance compensation control (UCC) method, using a general band-pass filter (GPF) to make the MLR spin around its geometric axis is also discussed. Experimental results show that the novel balancing method can remove more than 92.7% of the rotor unbalance and a balancing accuracy of 0.024 g mm kg^−1^ is achieved.

## 1. Introduction

Active magnetic bearings (AMBs) have several advantages over traditional bearings, such as low power losses, very long life, the elimination of the oil supply, vibration control, and diagnostic requirements, hence, AMBs have been widely used in the fields of energy-storing flywheels, turbo machinery and machine tools [1,2].

Due to material non-homogeneity and manufacturing errors, a rotor's inertia axis always misaligns with the geometric axis. This will inevitably result in rotor unbalance and produce centrifugal forces while the rotor is spinning. These centrifugal forces then transfer to the motor casing and generate vibration noises, which reduce the life of the machinery [3]. With respect to a magnetic levitated rotor (MLR), the rotor unbalance can even result in saturation of the magnetic actuator and lead to instability in the AMB control system [4].

Off-line balancing is a widely used method to eliminate rotor unbalance [5]. However, due to the limited precision of the balancing machines and the restrictions when changing from a balancing machine to the real working conditions, off-line balancing leaves considerable residual unbalances [6]. Based on the AMB's active control abilities, much research has focused on the active vibration control (AVC) method for an MLR, including the notch filter method [7], generalized notch filter (GNF) method [8], least mean square (LMS) algorithm [9], double-loop compensation method [10,11], *etc.* These methods suppress the vibrations produced by the synchronous current, and control the rotor to rotate around its inertia axis. The double-loop compensation method even suppresses the vibrations produced by negative position stiffness [11]. However, the AVC method also has several shortcomings. First, it cannot simultaneously realize zero-vibration and the zero-displacement, which is essential in fields such as molecular pumps. Second, the AVC method must be active while the machine is working, which requires rigorous stability and robustness of the algorithm.

The field balancing method employs correction masses to correct a rotor's mass distribution. It makes the rotor's inertia axis align with its geometric axis, removing the unbalance disturbance from the source [12]. It can simultaneously realize zero-vibration and zero-displacement. After balancing, there is no synchronous force between the rotor and the stator, and no vibration is transferred to the motor base. Meanwhile, the current consumption of AMBs will also be greatly reduced. Theoretically, a low-speed balancing can endow a rotor with balance throughout low-speed rotors, where the rigid rotor assumption is valid [13]. In addition, no particular control method is required after field balancing. Thus, field balancing is a one-time correction suitable for those rotors whose unbalance changes little during operation, such as energy-storing flywheels and vacuum pumps.

In recent years, various field balancing methods have been developed. These methods fall into two major categories: influence coefficient methods and modal balancing methods [14,15]. The influence coefficient methods require no assumptions other than the linearity of the rotor system and the measuring system. Thus, this method is well suited to field balancing and can achieve nearly ideal performance [16]. However, it has some unavoidable limitations, such as requiring a large number of test-runs, as influence coefficients are affected by rotating speeds [17]. Modal balancing methods separate the rotor vibration into a series of mode components. With the premise of learning the mode shape, only a single trial-run is required to gain the modal imbalance response. A full-speed range balance can be achieved after implementing balancing for all modal imbalances, but test-runs are indispensable. To overcome these limitations, several analytical methods, without trial weights, have been proposed in the recent literature [18,19]. Analytical methods have the advantage of requiring no trial runs, but they need to calibrate the rotor models very well, which is often not possible in industrial applications. Particularly for the AMB rotor, it is very difficult to establish an ideal mathematical model because of unmodelled dynamics, nonlinearities, and parameter uncertainties.

Displacement sensor and angular-position sensor permit field balancing for MLR can be implemented without any additional instrumentation. More importantly, AMBs have the ability to actively control the rotor. Nevertheless, there is little research on field balancing for MLR in particular. Li *et al.* [20] and Zhang *et al.* [21] used the influence coefficient method to perform field balancing for a MLR. Han *et al.* [22] introduced the analytical method to MLR, obtaining the equivalent static and dynamic imbalances, respectively, by detectng the translations signal and the rotational signal of the rotor. They did not however overcome the shortcomings of conventional balancing methods of being either inefficient or imprecise.

Based on an AMB rotor's active control property, this paper proposes a novel field balancing method. The method can simultaneously meet the requirements of high-efficiency and high-accuracy. It requires neither trial runs nor the MLR's precise model. In this method, the control current rather than rotor displacement is employed to calculate the correction masses, and no influence coefficients are required. After analysing the models of an unbalanced MLR, we find that the coincidence of a rotor's rotation axis with its geometric axis will bring about two benefits. One is that the unbalanced centrifugal force/torque equals the synchronous magnetic force/torque generated by the control current, which enables computation of correction masses using the control current with only a single start-up. The other is that the magnetic force is proportional to the control current, which makes the balancing highly accurate. The unbalance compensation control (UCC) method using a general band-pass filter (GPF), which enables the MLR to spin around its geometric axis, is also discussed.

## 2. Model of MLR Including Unbalance

As the first bending-critical speed (650 Hz) is far beyond the balancing speed (80 Hz), the rotor can be regard as peer rigid. All models in this study are based on this assumption.

### 2.1. Description of MLR System

**Figure 1.**
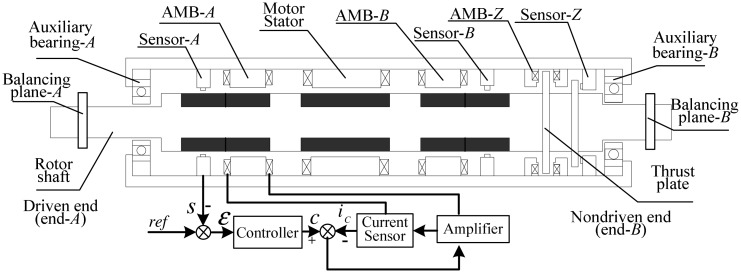
Structure diagram of a MLM.

The main structure of the magnetic levitation motor (MLM) is a motor which drives a rigid rotor supported by three AMBs, one thrust bearing and two radial bearings (see the computer-aided design diagram in Figure 1). The AMBs and displacement sensors are non-collocated. In addition, two balancing planes are placed at both ends of the rotor.

The AMB works in differential mode. Let *f_ax_* denote the magnetic force of the *AX*-channel (other channels are similar to *AX*). We then have:

(1) $$f_{ax}=K\left[\frac{{\left(I_{0}+i_{c}\right)}^{2}}{{\left(S_{0}-s\right)}^{2}}-\frac{{\left(I_{0}-i_{c}\right)}^{2}}{{\left(S_{0}+s\right)}^{2}}\right]$$

where *I*_0_ is the bias current, *i_c_* the control current, *S*_0_ the normal air gap, *s* the distance of rotor deviation from the magnetic center, and *K* is a characteristic constant of the electromagnet [1].

**Figure 2.**
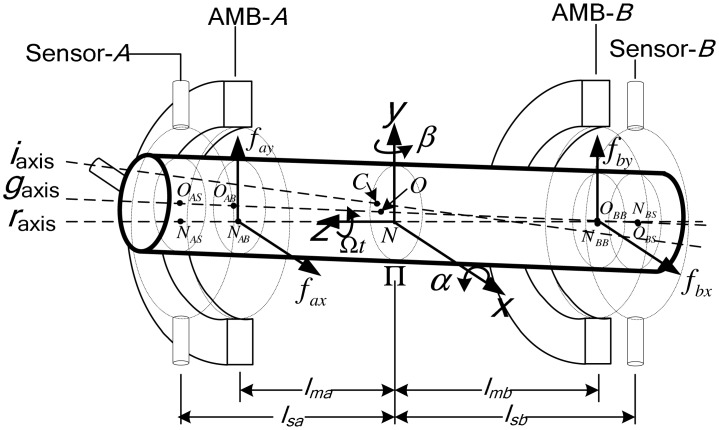
Schematic of an unbalanced rotor supported by two radial AMBs.

To intuitively model the unbalanced rotor, an unbalanced MLR supported by two radial AMBs is discussed here (Figure 2, referring to [23], but with two added sensor planes). Let *C* denote the rotor's mass center, residing in the plane Π. The points *O* and *N* are the geometric center and rotation center of Π. The axes *i*_axis_, *g*_axis_ and *r*_axis_ are the rotor's inertia axis, geometric axis, and rotation axis.

We establish the ground reference frame *Oxy*, where *x*-axis points to the *x* pole shoe of AMB*-A*. Let *α*, *β*, *Ùt* denote the angles of the rotor spinning around *Nx*, *Ny*, *Nz*, respectively. *l_sa_* and *l_sb_* (respectively, *l_ma_* and *l_mb_*) are the distances from *C* to the respective action planes of the two displacement sensors (respectively, two radial AMBs); *f_ax_*, *f_ay_*, *f_bx_*, *f_by_* are the magnetic forces of the two radial AMBs in *x* and *y* directions.

The unbalanced force is generated by the deviation of the inertia axis and rotation axis, which is divided into two parts. One is the deviation of the mass center and the rotation center, resulting in static unbalance. The other is the angle deviation of the inertia axis and rotation axis, resulting in dynamic unbalance.

### 2.2. Static Unbalance and Dynamic Unbalance

**Figure 3.**
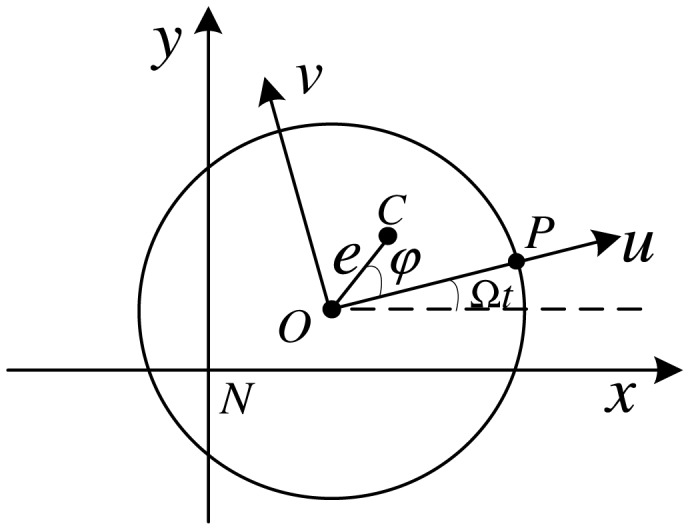
MLR's rotation center, geometric center and mass center in ground and rotor-fixed reference frame.

Static unbalance causes an unbalance force. The relative positions of the rotation center, geometric center and mass center when the rotor spins are shown in Figure 3.

The positions of the geometric center and mass center in *Nxy* are (*X*, *Y*) and (*x*, *y*). Point *P* is the absolute position measured by the Hall sensor. We establish the rotor-fixed reference frame *Ouv*, where the *u*-axis coincides with *OP. e* and *φ* denote the modulus and the angular position of the mass center *C* in *Ouv*, respectively. We have the following two relations about the static unbalance:

(2) $$\left\{ \begin{array}{l} e\times \cos(\Omega t+\varphi )=x-X\\ e\times \sin(\Omega t+\varphi )=y-Y \end{array}\right.$$

**Figure 4.**
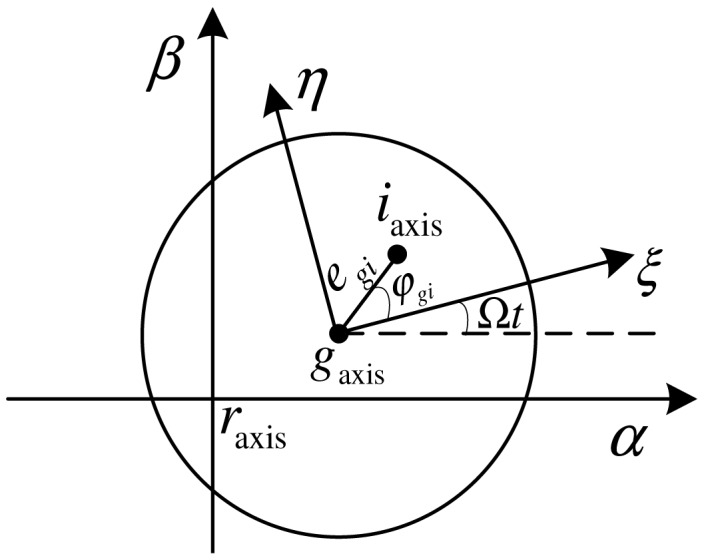
MLR's rotation axis, geometric axis and inertia axis in the ground and rotor-fixed angular coordinate system.

The rotor's dynamic unbalance causes an unbalance torque. Figure 4 represents the relative angular positions of axis *g*_axis_, *r*_axis_ and *i*_axis_, where *r*_axis_*αβ* is the static angular coordinate system and *g*_axis_*ξη* is the rotor-fixed angular coordinate system. Here, the angular coordinate system is employed to describe the relative angular position between the two axes. In the angular coordinate system, the position of an axis is determined by the included angle between this axis and the origin axis in directions of the two coordinate axes. (*α_rg_*,*β_rg_*) and (*α_ri_*,*β_ri_*) are the values of *g*_axis_ and *i*_axis_ in *r*_axis_*αβ*. Let *e_gi_* and *φ_gi_* denote the angular modulus and the initial angle (respect to *g*_axis_ in *ξ* direction) of *i*_axis_ in *g*_axis_*ξη*. The following relations about dynamic unbalance are gained:

(3) $$\left\{ \begin{array}{l} e_{gi}\cos(\Omega t+\varphi_{gi})=\alpha_{ri}-\alpha_{rg}\\ e_{gi}\sin(\Omega t+\varphi_{gi})=\beta_{ri}-\beta_{rg} \end{array}\right.$$

### 2.3. Model of the Unbalanced MLR

**Figure 5.**
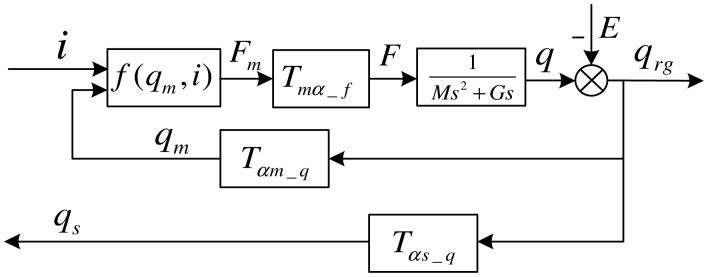
The control block diagram of the MLR.

For the purposes of this paper, the rotor is modeled as rigid. The control block diagram of the MLR system is shown in Figure 5. It is assumed that the vector *q*=[*x α y β*]*^T^* indicates translations and transverse rotations of the rotor's inertia axis about Point *N*, the vector *q_rg_*=[*X α_rg_Y β_rg_*]*^T^* indicates translations and transverse rotations of rotor's geometrical axis about Point *N*, *E* = [*e*cos(Ω*t*+ *φ*)*e_gi_*cos(Ω*t*+*φ_gi_*) *e*sin(Ω*t*+*φ*) *e_gi_* sin(Ω*t*+*φ_gi_*)]*^T^*. indicates the rotor's static and dynamic unbalance in the ground reference frame. Then, the dynamic equation for the rotor supported by magnetic bearings is:

(4) $$M\ddot{q}+\Omega G\dot{q}=F$$

The generalized mass matrix **M**, the gyroscopic matrix **G** and the generalized force vector *F* are defined as:

$$M=\left[\begin{array}{llll} m & 0 & 0 & 0\\ 0 & J_{r} & 0 & 0\\ 0 & 0 & m & 0\\ 0 & 0 & 0 & J_{r} \end{array}\right],G=\left[\begin{array}{llll} 0 & 0 & 0 & 0\\ 0 & 0 & 0 & -J_{z}\\ 0 & 0 & 0 & 0\\ 0 & J_{z} & 0 & 0 \end{array}\right],F=\left[\begin{array}{c} f_{x}\\ p_{x}\\ f_{y}\\ p_{y} \end{array}\right]$$

where *m* is the rotor mass, *J_r_* is the transverse moment of inertia, *J_z_* is the axial moment of inertia, *f_x_* and *f_y_* are the resultant forces in directions *Nx* and *Ny*, *P_x_* and *P_y_* are the moments about *Ny* and *Nx.*

The AMBs only provide forces at the locations indicated in Figure 2, and we define the magnetic force vector *F_m_* = [*f_ax_f_bx_f_ay_f_by_*]^T^. *F* can be expressed with *F_m_*:

(5) $$F=T_{m\alpha \_f}F_{m}$$

where $T_{m\alpha \_f}=\left[\begin{array}{cccc} 1 & 1 & 0 & 0\\ l_{ma} & -l_{mb} & 0 & 0\\ 0 & 0 & 1 & 1\\ 0 & 0 & -l_{ma} & l_{mb} \end{array}\right]$ is the force transformation matrix. According to Equation (1) *f_ax_*, *f_bx_*, *f_ay_*, *f_by_* can be expressed as:

(6) $$\{\begin{array}{c} f_{ax}=k\left[\frac{{\left(I_{0}+i_{ax}\right)}^{2}}{{\left(s_{0}-x_{ax}\right)}^{2}}-\frac{{\left(I_{0}-i_{ax}\right)}^{2}}{{\left(s_{0}+x_{ax}\right)}^{2}}\right]\\ f_{bx}=k\left[\frac{{\left(I_{0}+i_{bx}\right)}^{2}}{{\left(s_{0}-x_{bx}\right)}^{2}}-\frac{{\left(I_{0}-i_{bx}\right)}^{2}}{{\left(s_{0}+x_{bx}\right)}^{2}}\right]\\ f_{ay}=k\left[\frac{{\left(I_{0}+i_{ay}\right)}^{2}}{{\left(s_{0}-x_{ay}\right)}^{2}}-\frac{{\left(I_{0}-i_{ay}\right)}^{2}}{{\left(s_{0}+x_{ay}\right)}^{2}}\right]\\ f_{by}=k\left[\frac{{\left(I_{0}+i_{by}\right)}^{2}}{{\left(s_{0}-x_{by}\right)}^{2}}-\frac{{\left(I_{0}-i_{by}\right)}^{2}}{{\left(s_{0}+x_{by}\right)}^{2}}\right] \end{array}$$

We denote *q_m_*=[*x_ax_x_bx_x_ay_x_by_*]*^T^* and *i*=[*i_ax_i_bx_i_ay_i_by_*]*^T^* as rotor vibration vector at bearings and coil current vector. Then, *F_m_* can be described as a non-linear function:

(7) $$F_{m}=f(i,q_{m})$$

According to Equations (2) and (3), the relationship between *q* and *q_rg_* can be described as follows:

(8) $$q=q_{rg}+E$$

Substituting Equation (8) into Equation (4), dynamic equation of the unbalanced rotor becomes:

(9) $$M\left({\ddot{q}}_{rg}+\ddot{E}\right)+\Omega G\left({\dot{q}}_{rg}+\dot{E}\right)=F$$

When we describe the magnetic force produced by rotor vibration, it is necessary to transform from the gravity-center coordinates to the bearing coordinates since the rotor dynamics are formulated in the gravity-center coordinate system. The matrix *T_αm_*___*_q_* is used to realize the transform from *q_rg_* to *q_m_* (see Figure 5), defined as:

(10) $$T_{\alpha m\_q}=\left[\begin{array}{cccc} -1 & -l_{ma} & 0 & 0\\ -1 & l_{mb} & 0 & 0\\ 0 & 0 & 1 & -l_{ma}\\ 0 & 0 & 1 & l_{mb} \end{array}\right]$$

The sensors are non-collocated with the bearing center of action. To account for this, an additional transform is required to move from the sensors to the gravity center coordinate system.

(11) $$T_{\alpha s\_q}=\left[\begin{array}{cccc} -1 & -l_{sa} & 0 & 0\\ -1 & l_{sb} & 0 & 0\\ 0 & 0 & 1 & -l_{sa}\\ 0 & 0 & 1 & l_{sb} \end{array}\right]$$

## 3. Calculating the Correction Masses

For traditional supported rotors, knowing the rotor unbalance *E* is conducive to calculate the correction masses. However, *E* cannot be directly measured, whereas, *q_rg_* can be measured using the displacement sensors, the rotation angle Ω*t* can be measured by the Hall sensor. Therefore, some methods estimate *E* through *q_rg_*, assuming that their relationships are linear, e.g., influence coefficient method. However, several trial-runs of influence coefficient method make the field to be inefficient.

For a MLR, however, we can obtain the expression of *E* easily. The rotor's unbalance response is synchronous with the rotor speed. Thus, only the synchronous component in Equation (9) is of interest, and the unbalance can be solved as:

(12) $$E={\left(-\Omega^{2}M+j\Omega G\right)}^{-1}\left(F+\Omega^{2}Mq_{rg}-j\Omega^{2}Gq_{rg}\right)$$

**Figure 6.**
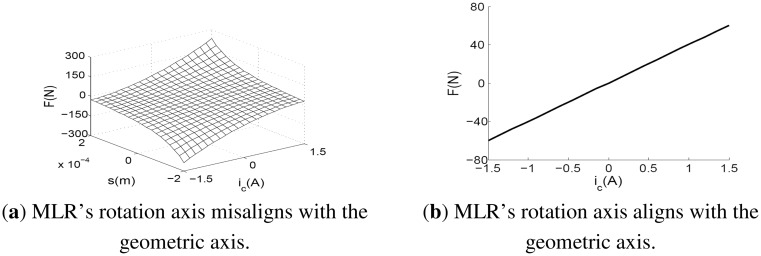
Force-current/displacement characteristic of the radial AMB.

When we use Equation (12) to solve the unbalance, *F* and *q_rg_* must be measured beforehand. In addition, it is found from Equation (7) that *F* is a function of *q_m_* and *i*, and the relationships between them are nonlinear (see Figure 6a). This procedure is complex, and it will produce a lot of calculation errors and nonlinear errors. When the rotor deviates heavily from the bearing centerline under a large unbalance, the nonlinear error cannot be ignored if high-accuracy balancing is demanded. Based on the MLR's characteristic of active control, here, we try to control the rotor operate under a state that can simplify the above process and possess a linear magnetic force.

**Figure 7.**
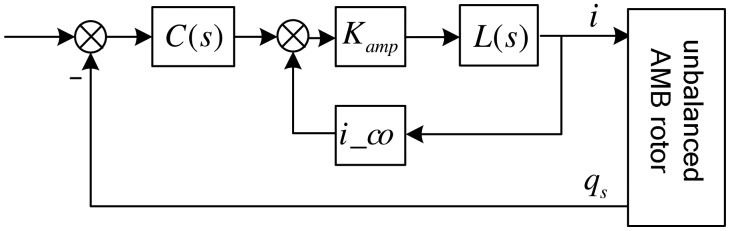
The control block diagram of the MLR system.

As the MLR's control diagram shown in Figure 7, the control current can be described as:

(13) $$i={\left[I+i_{c\_o}K_{\text{amp}}L(s)\right]}^{-1}K_{\text{amp}}L(s)C(s)q_{s}$$

where *q_s_*=T*_αs_*___*_q_q_rg_* is the vector of sensor output, *C*(*s*) is the controller, *L*(*s*) is the transfer function of the AMB coil, *i_co* and *K_amp_* the feedback-gain and forward-gain coefficients of the current control loop.

We know that the value of *i* is finite. If the synchronous gain of the controller *C*(s) can be adjusted to be infinite at the promise that the MLR system is stable, the synchronous component in *q*_s_ will reduce to be zero. *q_rg_* becomes to zero, too. We call the situation as the zero-displacement status, and the expression of the unbalance vector will be simplified as:

(14) $$E={\left(-\Omega^{2}M+j\Omega G\right)}^{-1}F$$

Because the transformation from *q*_m_ to *q*_s_ is linear (shown in Equations (10) and (11)), we gain *q_m_* = [0,0,0,0]^T^ at zero-displacement status. Substituting *q_m_* = [0,0,0,0] into Equation (6), the expression of *F*_m_ = [*f_ax_*, *f_bx_*, *f_ay_*, *f_by_*]^T^ can be simplified to:

(15) $$F_{m}=\left[\begin{array}{c} f_{ax}\\ f_{bx}\\ f_{ay}\\ f_{by} \end{array}\right]=\left[\begin{array}{c} \frac{4kI_{0}}{{\left(s_{0}\right)}^{2}}i_{ax}\\ \frac{4kI_{0}}{{\left(s_{0}\right)}^{2}}i_{bx}\\ \frac{4kI_{0}}{{\left(s_{0}\right)}^{2}}i_{ay}\\ \frac{4kI_{0}}{{\left(s_{0}\right)}^{2}}i_{by} \end{array}\right]=k_{i}i$$

in which *k_i_* = *4kI_0_/s_0_^2^* is a constant. Now, we obtain the other ideal result: electromagnetic force *F_m_* is proportional to the control current *i*, independent of the rotor's displacement *q_s_* (see Figure 6b). Substituting Equations (5) and (15) into Equation (14), we have:

(16) $$E={\left(-\Omega^{2}M+j\Omega G\right)}^{-1}T_{m\alpha \_f}k_{i}i$$

**Figure 8.**
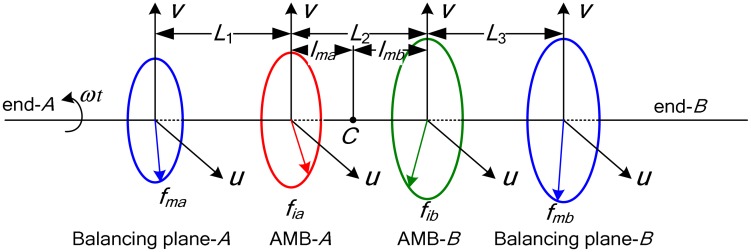
Schematic of the double-plane balancing method.

Theoretically, any unbalance distribution in a rigid rotor can be balanced in two different planes [24], which is the so-called double-plane balancing method. As illustrated in Figure 8, the inter-plane distances are denoted as *L*_1_, *L*_2_ and *L*_3_, and *L*_1_ + *L*_2_ + *L*_3_ = *L*.

Assuming that the correction masses *m_ca_* and *m_cb_* are placed at the azimuth angles *φ_ma_* and *φ_mb_* on the two balancing planes, the generalized correction vector can be defined as:

(17) $$M_{\alpha \_c}=T_{m\alpha \_c}m_{c}=\left[\begin{array}{cccc} 1 & 1 & & \\ \left(L_{1}+l_{ma}\right) & -(L_{3}+l_{mb}) & & \\ & & 1 & 1\\ & & -(L_{1}+l_{ma}) & \left(L_{3}+l_{mb}\right) \end{array}\right]\;\left[\begin{array}{c} m_{ca}r_{ca}\cos(\Omega t+\varphi_{ca})\\ m_{cb}r_{cb}\cos(\Omega t+\varphi_{cb})\\ m_{ca}r_{ca}\sin(\Omega t+\varphi_{ca})\\ m_{cb}r_{cb}\sin(\Omega t+\varphi_{cb}) \end{array}\right]$$

According to the double-plane balancing method, if *M*_c_ can balance the rigid rotor completely, the following formulation will be obtained:

(18) $$ME+M_{\alpha \_c}=0$$

For the slender rotor considered in this study, *J_r_* is significantly larger than *J_z_*, so the gyroscopic moment term in Equation (16) can be ignored, and then substitute Equation (16) into Equation (18), we have:

(19) $$M_{\alpha \_c}\Omega^{2}=T_{m\alpha \_f}k_{i}i$$

The correction masses can be solved from Equation (19), and the result is:

(20) $$M_{c}=\frac{1}{\Omega^{2}}{T_{m\alpha \_c}}^{-1}T_{m\alpha \_f}k_{i}i=\frac{1}{L\Omega^{2}}\left[\begin{array}{cccc} L_{2}+L_{3} & L_{3} & & \\ L_{1} & L_{1}+L_{2} & & \\ & & L_{2}+L_{3} & L_{3}\\ & & L_{1} & L_{1}+L_{2} \end{array}\right]k_{i}i$$

where *L*, *L*_1_, *L*_2_, *L*_3_ can be obtained precisely from the mechanical layout, rotor speed Ω can be measured by the Hall sensor. According to Equation (20), the unbalance *m_c_* will be calculated directly after obtaining the current stiffness *K_i_* and control current *i*. Compared to the field balancing before adding the infinite gain control, the formulate here need not *q*_m_, *q*_rg_, *l*_ma_ and *l*_mb_, and the magnetic force is the linear function of the rotor vibrations.

The correction masses on the two balancing planes can be solved further as:

(21) $$\left\{ \begin{array}{l} m_{ca}=\sqrt{{m_{au}}^{2}+{m_{av}}^{2}}\\ m_{cb}=\sqrt{{m_{bu}}^{2}+{m_{bv}}^{2}}\\ \varphi_{ca}=\text{arctan}\left(\frac{m_{av}}{m_{au}}\right)\\ \varphi_{cb}=\text{arctan}\left(\frac{m_{bv}}{m_{bu}}\right) \end{array}\right.$$

where:

(22) $$\{\begin{array}{c} m_{au}=\left[k_{\text{iax}}i_{au}(L_{2}+L_{3})+k_{\text{ibx}}i_{bu}L_{3}\right]/\left(r_{a}\Omega^{2}L\right)\\ m_{av}=\left[k_{\text{iax}}i_{av}(L_{2}+L_{3})+k_{\text{ibx}}i_{bv}L_{3}\right]/\left(r_{a}\Omega^{2}L\right)\\ m_{bu}=\left[k_{\text{iax}}i_{au}L_{1}+k_{\text{ibx}}i_{bu}(L_{1}+L_{2})\right]/\left(r_{b}\Omega^{2}L\right)\\ m_{bv}=\left[k_{\text{iax}}i_{av}L_{1}+k_{\text{ibx}}i_{bv}(L_{1}+L_{2})\right]/\left(r_{b}\Omega^{2}L\right) \end{array}$$

Field balancing under the zero-displacement status greatly improves calculation accuracy and reduces the computation process, requiring only a single start-up. In addition, rigid balancing just needs to be implemented at a low speed. The magnetic force is small in this case, and the AMB saturation will not take place, ensuring the magnetic force is in proportion to the control current.

## 4. Unbalance Compensation Control

The advantages of field balancing under the zero-displacement status have been discussed in detail in the section above. In this section, we will discuss how to control the rotor to rotate under this status.

It is known that the object of active vibration control is to make the rotor spin around its inertia axis, which requires the synchronous magnetic force between rotor and stator to be infinitesimal (equivalent to the synchronous support-stiffness being infinitesimal). Under the zero-displacement status, the rotor is forced to spin around its geometric axis. Its synchronous displacement is infinitesimal whereas the synchronous support-stiffness is infinite, a situation also called unbalance compensation control method.

**Figure 9.**
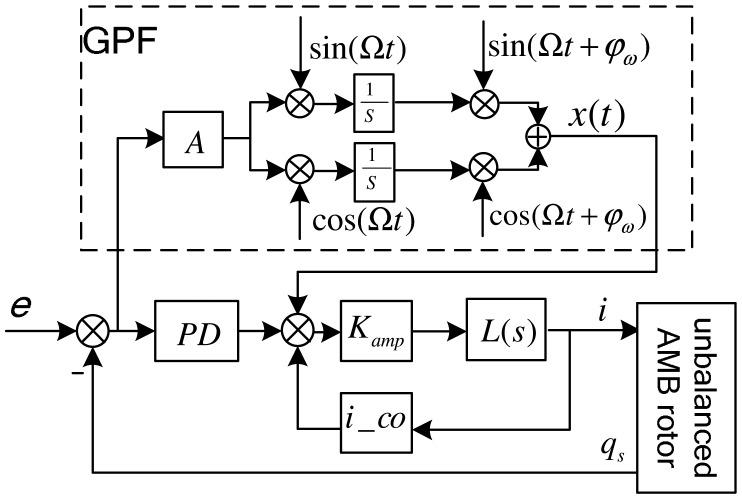
AMB control system including the GPF.

Field balancing described in this study is realized at a fixed speed, without guaranteeing the stability of the closed loop over the full-speed range, but requiring a high-accuracy algorithm. Thus, referring to the GNF discussed by Hezorg *et al.* [8], a generalized band-pass filter (GPF) is proposed to make the AMB controller's synchronous-gain infinite. The diagram of the AMB control system including the GPF is shown in Figure 9, where *A* is the gain coefficient of the GPF.

**Figure 10.**
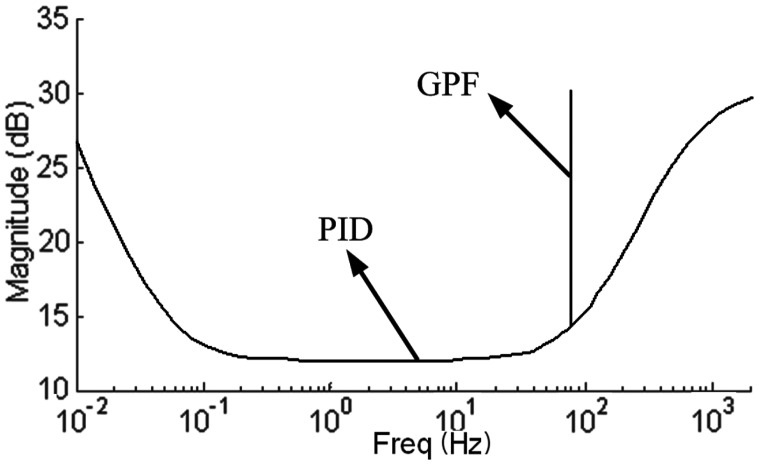
Amplitude-frequency response characteristic of the controller (GPF + PD).

As the gyroscopic coupling of the slender rotor is weak, an SISO controller woke well. And the stability analysis is based on SISO controller. In Figure 9, we use a proportional-derivative (PD) controller to levitate the rotor. The GPF is in parallel with the PD controller, supplying a synchronous infinite gain to the PD controller (see the amplitude-frequency response characteristic of the controller shown in Figure 10).

The GPF is briefly described as follows. First, the cross-correlation coefficients between the control error signal *e*(*t*) and synchronous sin/cos signals are obtained by the correlation calculation:

(23) $$\left\{ \begin{array}{l} a=\frac{2}{T}\int_{t_{0}}^{T+t_{0}}e(t)\sin(\Omega t)dt\\ b=\frac{2}{T}\int_{t_{0}}^{T+t_{0}}e(t)\cos(\Omega t)dt \end{array}\right.$$

Then; the synchronous component of the error signal is reconstructed:

(24) x(t)=A⋅(a⋅sinΩt+b⋅cosΩt)

The synchronous component passes through integrators until it vanishes, and a constant synchronous control output is achieved through GPF channel. The control output cancels out the unbalance forces.

To ensure the stability margin of the GNF, a transform matrix was strung behind the integral part [8]. Because the gyroscopic coupling of our MLR is weak, the transform matrix can be replaced by a phase correction *φ_ω_*, which simplifies the correction from a matrix to a variable. Thus, Equation (24) is converted to:

(25) $$x(t)=A\cdot \left(a\cdot \sin(\Omega t+\varphi_{\omega })+b\cdot \cos(\Omega t+\varphi_{\omega })\right)$$

Herzog utilized the root locus to analysis the GNF's stability [8]. However, the parameters' exact stable region was not obtained in his study. For the SISO case, Li *et al.* [10] assume a constant Ω, but use a Bode plot to give a stability property, which was straightforward and clarified the stability robustness against unknown high-frequency dynamics in *H*(s). However, their analysis about stability was established at the promise that *A* was arbitrarily small, which results in slow convergence. To simplify the stability analysis, Tang *et al.* [11] eliminated the transform matrix in GNF, which makes the GNF unstable near the critical speed.

**Figure 11.**
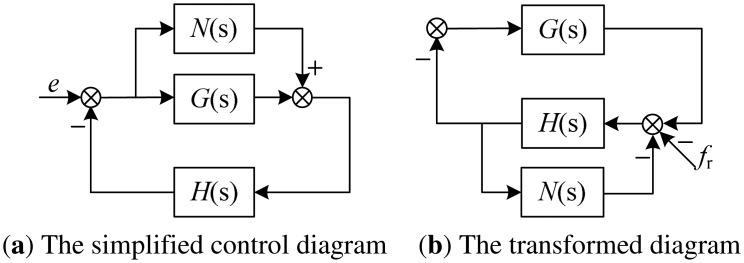
The simplified control diagram and its transformation of the MLR.

Following the analysis of GNF, the stability of the GPF will be discussed here. However, unlike the GNF, the stable region of the GPF will be solved exactly in this study. First, the AMB control system described in Figure 9 is simplified as shown in Figure 11a, in which the additional controller is set as *N*(s), the original controller is set as *G*(s), the controlled object including AMB and the rotor is set as *H*(s), *e* is the unbalance. Then, the control structure in Figure 11a is easily transformed into the structure in Figure 11b, in which the unbalance is equivalent to *f_r_*, *N*(s) is equivalent to an inside feedback of the controlled object.

*N*(s) also can be regarded as the model error of the controlled object *H*(s). Then, the actual controlled object is *H*(*s*)/[1+*H*(*s*)*N*(*s*)]. The model error described parallel to *H*(s) is:

(26) $$\Delta (s)=\frac{H(s)}{1+H(s)N(s)}-H(s)=-\frac{H(s)H(s)N(s)}{1+H(s)N(s)}$$

Using the small gain theorem [25], the stability boundary for the system is:

(27) $$\left\|H_{cl}(j\omega )\Delta (j\omega )/H(j\omega )\right\|<1$$

That is:

(28) $$\left\|\frac{H(j\omega )G(j\omega )H(j\omega )N(j\omega )}{\left\{1+H(j\omega )\left[G(j\omega )+N(j\omega )\right]+H(j\omega )G(j\omega )H(j\omega )N(j\omega )\right\}}\right\|<1$$

It is found from Equation (28) that the control system is critical stable when *N*(j*ω*) satisfies:

(29) 1+H(jω)[G(jω)+N(jω)]=0

Substituting system parameters and the expression of *N*(s) defined in [8] to Equation (29), we have:

(30) $$1+\frac{1}{m{\left(j\omega \right)}^{2}-K_{h}}\times \frac{k_{\text{amp}}}{L\times j\omega +R+i\_co\times k_{\text{amp}}-K_{h}}\times \left[\left(k_{p}+k_{d}\times j\omega \right)+\frac{A_{C}\times j\omega +A_{S}\times \Omega }{{\left(j\omega \right)}^{2}+\Omega^{2}}\right]=0$$

where *A*_C_ = *A*cos(*φ_ω_*), *A*_S_ = *A*sin(*φ_ω_*), *k*_p_ and *k*_d_ are proportionality and differential coefficient.

Equation (30) can be separated into the real part equation and the imaginary part equation:

(31) $$\left\{ \begin{array}{l} \left[Lm\times {\left(j\omega \right)}^{3}+(k_{\text{amp}}k_{d}-Lk_{h})\times j\omega \right]({\left(j\omega \right)}^{2}+\Omega^{2})+A_{C}\times j\omega =0\\ \left[\left(R+i\_co\times k_{\text{amp}}\right)M\times {\left(j\omega \right)}^{2}-k_{h}\left(R+i\_co\times k_{\text{amp}}\right)+k_{\text{amp}}k_{p}\right]({\left(j\omega \right)}^{2}+\Omega^{2})+A_{S}\times \Omega =0 \end{array}\right.$$

Solving the above equations, *A*_c_ and *A*_s_ are obtained as:

(32) $$\left\{ \begin{array}{l} A_{c}=\left[LM\omega^{2}+Lk_{h}-k_{\text{amp}}k_{d}\times k\_i\times k_{s}\right](\Omega^{2}-\omega^{2})/(k\_i\times k_{s})\\ A_{s}=-\frac{\Omega^{2}-\omega^{2}}{\Omega }\left[k_{\text{amp}}k_{p}\times k\_i\times k_{s}-\left(R+i\_co\times k_{\text{amp}}\right)\left(M\omega^{2}+k_{h}\right)\right]/(k\_i\times k_{s}) \end{array}\right.$$

The expression of *ω*^2^ can be gained from Equation (32):

(33) $$\omega^{2}=\frac{A_{C}(R+i\_co\times k_{\text{amp}})-A_{S}L\Omega }{k_{\text{amp}}k_{d}\left(R+i\_co\times k_{\text{amp}}\right)-Lk_{\text{amp}}k_{p}}+\Omega^{2}$$

**Figure 12.**
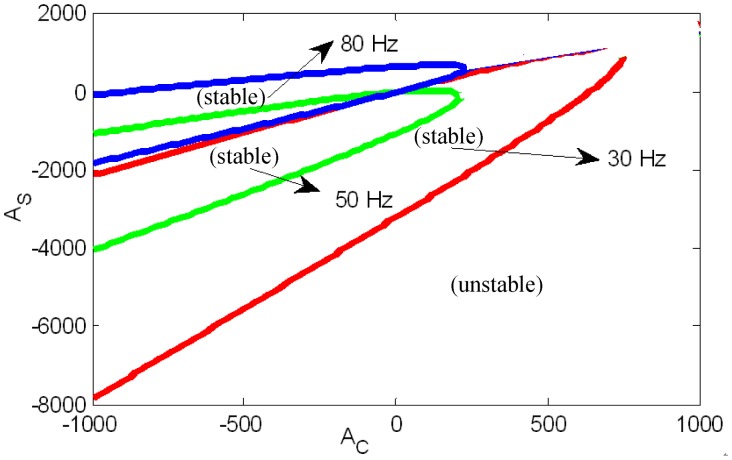
Parameters *A_C_* and *A_S_* satisfying the critical stability under the different speed.

Substituting Equation (33) to Equation (32), the relationship between *A*_c_, *A*_s_ and Ω can be solved. Parameters *A*_c_ and *A*_s_ that satisfy the critical stability under the different rotor speeds are shown in Figure 12. We can see that: (1) stable regions under different speeds are all passing through the origin of coordinates; (2) the stable regions of the low speed and high speed have no intersection, and the low speed and the high speed are separated by the rigid critical speed.

**Figure 13.**
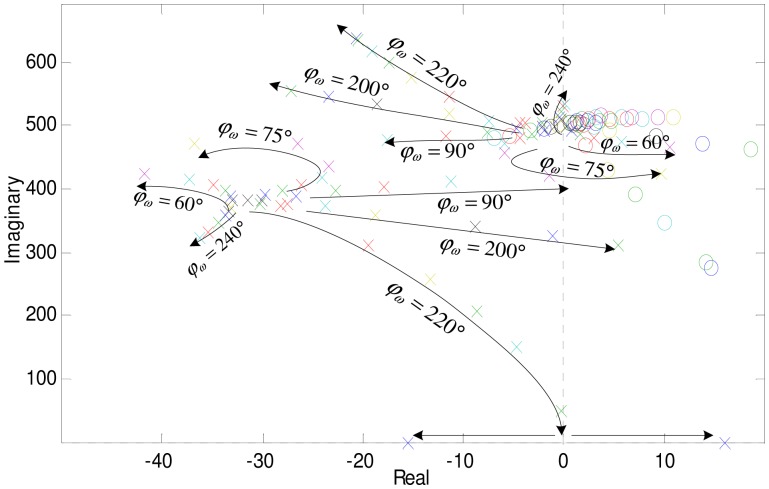
Poles and zeros of the AMB system with GPF at speed of 80 Hz.

Figure 13 describes that the poles of the close-loop system changes with GPF control parameters at the speed of 80 Hz (only the poles above the real axis are displayed in the figure, as they are symmetrical about the real axis). Control parameters in the simulations are the same as those used in experiments. The gain coefficient *A* and phase corrector *φ_ω_* are two important parameters of the GPF. Curves in Figure 13 describe the trend of the close-loop system poles when increasing *A* under the same *φ_ω_*.

Any pole falling to the right-half plane will make the AMB control system unstable. There are two poles in Figure 13. One is the original system's pole, we call it S-pole. To make the original system stable, the S-pole should locate in the left half plane. The other pole is produced by the GPF, we call it GPF-pole. The GPF-pole locates on the imaginary axis with an initial value of Ω. As shown in the figure, the S-pole changes with the GPF. With parameter *A* increasing, the S-pole even shifts to the right-half plane. Thus, the system with GPF has three instability forms: (1) GPF-pole shifts to the right-half plane directly; (2) S-pole shifts to the right-half plane directly; (3) S-pole shifts to the right-half plane passing through the real-axis. The first two are in accordance with the stable region displayed in Figure 12, while the third should be solved according to Equation (33). When *φ_ω_* = 220°, the trending curves just right pass near the original point. The S-poles first join at the real-axis and then separate along the real-axis, one of which falls to the right-half plane.

**Figure 14.**
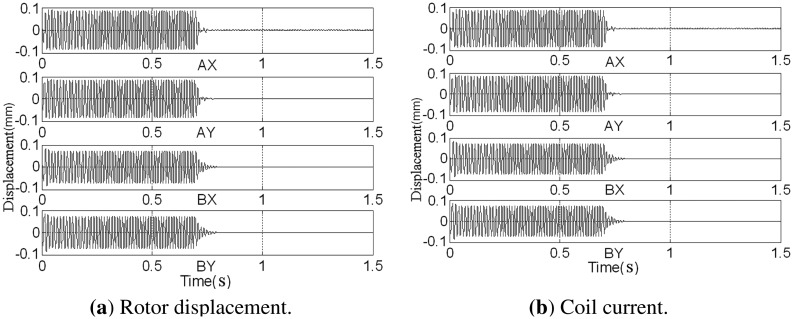
Signals before and after adding the GPF (simulation performed with Simulink).

The GPF will make the rotor displacement converge to zero as long as *A*_S_ and *A*_C_ lie in the stable region in Figure 12. However, to improve the convergence speed, *A* should not be enlarged limitlessly but the real parts of S-pole and GPF-pole should be decreased. Here, *φ*_ω_ is set 140°, and *A* is set 350°. The simulation results are shown in Figure 14. After adding the GPF, the MLR's rotation axis quickly converges to the geometric axis.

## 5. Measuring the Magnetic Force

As discussed in Section 3, the procedure of computing the correction masses will be simplified greatly when the rotor is controlled under the zero-displacement status. Nevertheless, control current and current stiffness are indispensable, and the two factors will directly influence the computational precision. Accurate acquisition of the above two factors will be discussed in this section.

### 5.1. Measuring Current Stiffness

Due to manufacturing and assembling errors, the current stiffness is usually not the same as its design value, and balancing accuracy will decrease when using the design value. Because the geometric axis aligns with the AMBs centerline at zero-displacement status, the current stiffness loss produced by eddy current can be ignored in low-speed filed balancing. Here, torque equilibrium method, a simple but useful method, is employed to test the current stiffness.

**Figure 15.**
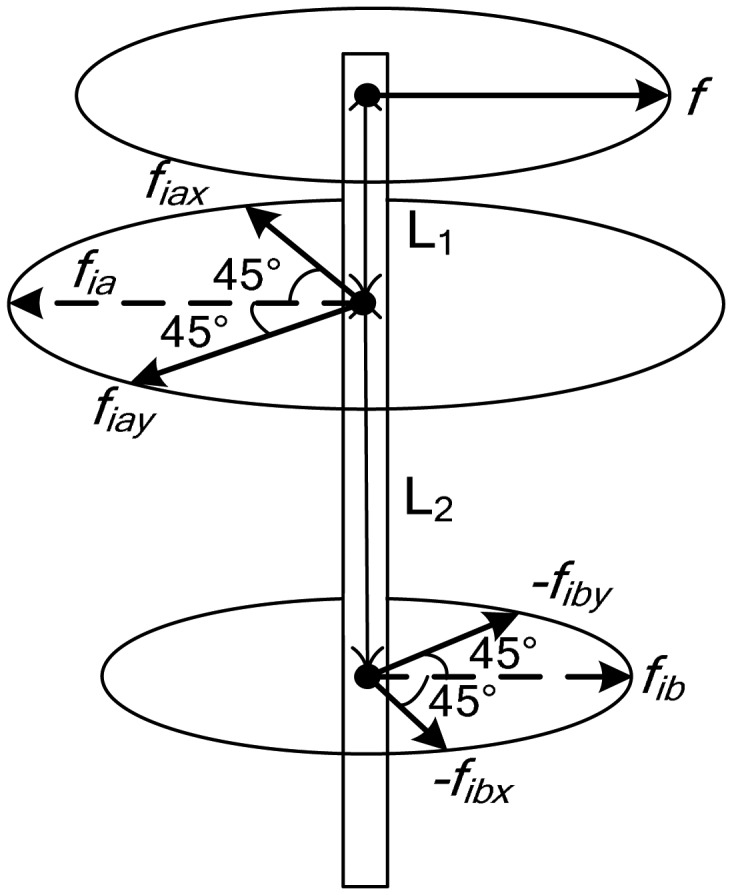
Schematic of current stiffness measurement.

As shown in Figure 15, the MLM is placed vertically, and the rotor is levitated along the bearing centerline. A force *f*, supplied by a spring balance, is added at balancing plane-*A* and point to the middle of AMB-*A*'s two pole shoes. The torque equilibrium equations for the rotor are:

(34) $$\left\{ \begin{array}{l} \sqrt{2}/2f\times (L_{1}+L_{2})\times (1+j)+(f_{\text{iax}}+j\times f_{\text{iay}})\times L_{2}=\sqrt{2}/2f\times (L_{1}+L_{2})\times (1+j)+(k_{\text{iax}}i_{ax}+j\times k_{\text{iay}}i_{by})\times L_{2}=0\\ \sqrt{2}/2f\times L_{1}\times (1+j)-(f_{\text{ibx}}+j\times f_{\text{iby}})\times L_{2}=\sqrt{2}/2f\times L_{1}\times (1+j)-(k_{\text{ibx}}i_{bx}+j\times k_{\text{iby}}i_{by})\times L_{2}=0 \end{array}\right.$$

From Equation (34), the current stiffness can be solved as:

(35) $$\left\{ \begin{array}{l} k_{\text{iax}}=-f\times (L_{1}+L_{2})/(\sqrt{2}L_{2}i_{ax})\\ k_{\text{iay}}=-f\times (L_{1}+L_{2})/(\sqrt{2}L_{2}i_{ay})\\ k_{\text{ibx}}=-f\times L_{1}/(\sqrt{2}L_{2}i_{bx})\\ k_{\text{iby}}=-f\times L_{1}/(\sqrt{2}L_{2}i_{by}) \end{array}\right.$$

### 5.2. Extracting the Synchronous Current

Because unbalance is a rotor's feature, the unbalance disturbance force is synchronized with the rotation rotor. Thus, the correction masses in Equation (17) are defined in a rotor-fixed coordinate system. However, coil currents are measured in the ground coordinate system because the AMB coils are fixed in the stator. The current frequency produced by unbalance is the same as the rotor rotation frequency. Beside the signals produced by unbalance disturbances, the measured current is mixed with signals with other frequencies. Therefore, to obtain the correction masses, we should extract the component that is synchronized with the rotation speed from the measured current, and then describe it in a rotor-fixed coordinate system through coordinate conversion. The cross-correlation algorithm (CCA) [26] can simultaneously realize the above two steps: extracting the synchronous signal from the measured current and accomplishing coordinate conversion. In the CCA, the measured current *i*(*nT*) is performed correlation operating with sin(*nT*) and cos(*nT*) respectively:

(36) $$\left\{ \begin{array}{l} i_{a}=\frac{2}{NT}\sum_{n=1}^{N}\left[i(nT)\cos\_\text{tab}(n\frac{N_{z}}{N})\right]\\ i_{b}=\frac{2}{NT}\sum_{n=1}^{N}\left[i(nT)\sin\_\text{tab}(n\frac{N_{z}}{N})\right] \end{array}\right.$$

where *N*_z_ is the size of the sine/cosine tables, *N* is the rotational period, *T* is the sampling time. *N* changes with the rotation speed, and the frequency of sine/cosine series will change accordingly. The two results are called the cross-correlation coefficients, which are the coordinate values of the current produced by the unbalance in the rotor-fixed coordinate frame.

## 6. Platform Introduction and Experimental Results

The rated speed (1,000 Hz) of the rotor in the study is higher than its first bending frequency (650 Hz), so the rotor is flexible. It is necessary to implement flexible balancing. However, to make the flexible balancing more precise, the beforehand rigid balancing is indispensable. Rigid balancing is mainly discussed in this study. The rotor is rigid when it rotates below 50% of the first bending critical speed *f*_bending_, weakly rigid when above 50% and below 70% of *f*_bending_, and flexible when above 70% [27]. Theoretically, the rigid balancing can be carried out at any speed when the rotor is rigid, because at which the rigid unbalance plays a key role and the flexible influence can be ignored [13]. The AMB force in our study (the maximum is 50 N) cannot counterbalance the centrifugal force produced by the residual unbalance at high speed. Therefore, we implement rigid balancing at 80 Hz, and rotor will be balanced at full speed when the rotor is rigid.

### 6.1. Introduction of the Platform

**Figure 16.**
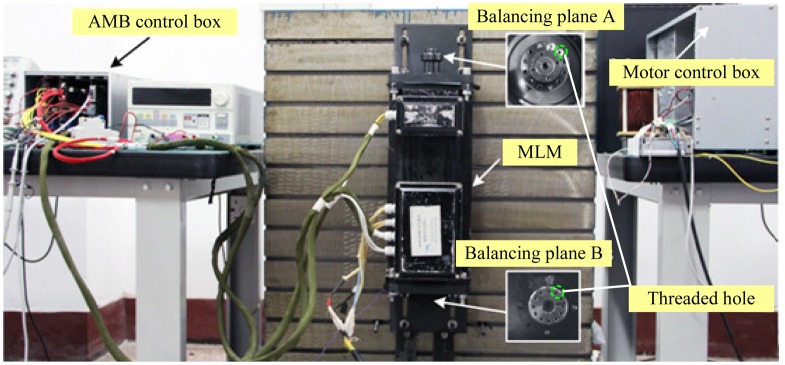
Photograph of the 4 kW MLM, AMB control box and motor control box.

To evaluate the effectiveness of the proposed balancing method, experiments were carried out on an MLM, which was fixed vertically on a metal base (as shown in Figure 16). The design parameters of this MLM are listed in Table 1. Two balancing planes are added to the rotor's both ends. The rotor is supported at the reference position by the AMB control box and is driven by the motor control box. AMB controller utilizes TMSF28335 as its compute unit, controller's sampling period is 150 μs.

The inertia axis and the rotation axis of the rotor are not aligned when rotating because of the residual unbalance. To reduce the unbalance, correction masses should be added to the two balancing planes at appropriate positions. There are 12 thread holes meanly distributing in every balancing plane. Screws matching the correction masses are fixed in the thread holes that match the correction angles. If the correction angle mismatches any thread hole, two screws that equivalent the correction masses can be placed in the two nearby thread holes.

### 6.2. Experimental Results

**Figure 17.**
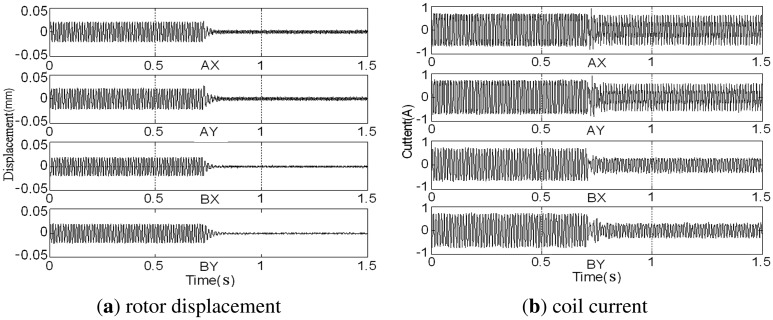
Sensor outputs before and after adding the GPF at 80 Hz.

Before assembled in the casing, the rotor has been balanced on the balancing machine. The GPF is added to the AMB control system at 0.7 s, and changes of the rotor displacement and coil current are shown in Figure 17.

**Figure 18.**
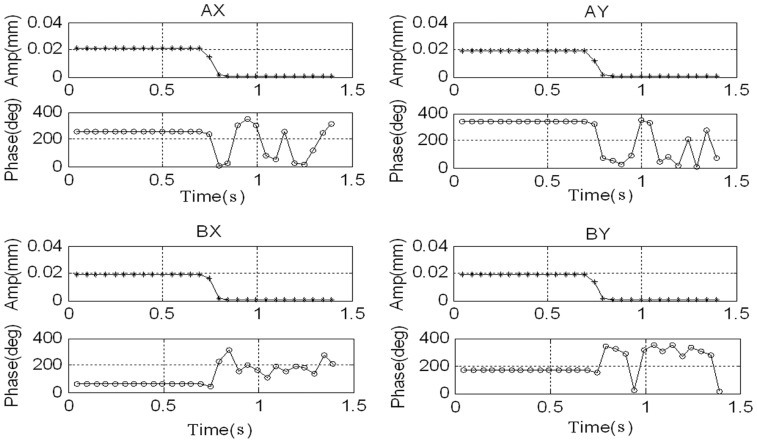
Amplitude and phase of synchronous displacement before and after adding the GPF.

**Figure 19.**
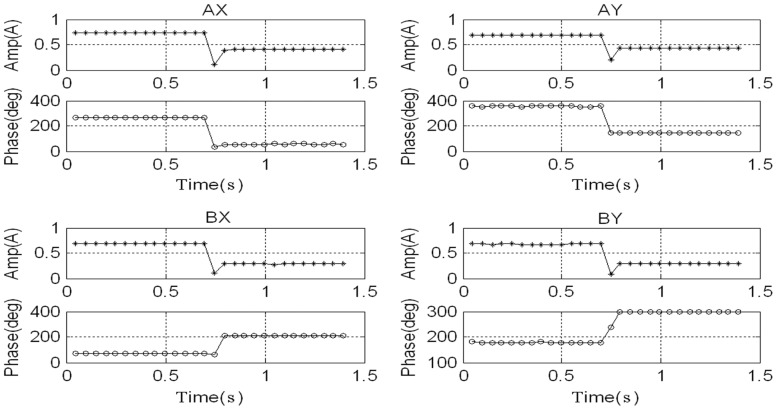
Amplitude and phase of synchronous current before and after adding the GPF.

The rotor's synchronous displacement rapidly decreases to zero (the high-frequency residuals of *AX*-channel and *AY*-channel are caused by the rough sensor target rather than the unbalance) after adding GPF. With the CCA, the amplitudes and phases of the synchronous displacement and current are extracted (see Figures 18 and 19). The amplitudes of *X*-channel and *Y*-channel are almost the same, whereas the phase of *Y*-channel leads that of *X*-channel by 90 as the rotor spins from the *x*-axis to the *y*-axis.

According to the method discussed in Section 5, the measured current stiffness are obtained and listed in Table 2, which are all below the design values.

According to Equation (21), the synchronous current of *X*-channel is employed to compute the correction masses. The original correction masses at both balancing planes are 1.292 g∠154.4° and 0.896 g∠264.0° respectively (equivalent to 3.772 g mm kg^−1^ and 1.962 g mm kg^−1^), far from the standard of balance grade for a motor [28]. After balancing once, the correction masses are 0.045 g∠185.5° and 0.063 g∠45.3°, reducing 97.5% and 92.7% respectively. After a second balancing, the residual unbalances are 0.011 g∠54.1° and 0.011 g∠126.8° (equivalent to 0.032 g mm kg^−1^ and 0.024 g mm kg^−1^), superior to the standard of the highest balance grade in ISO1940–2003 [28].

**Figure 20.**
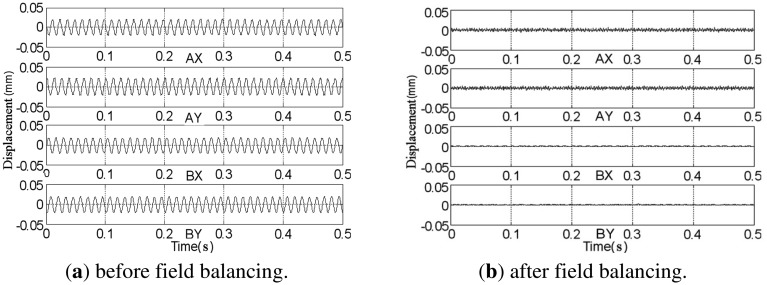
Rotor displacement at 80 Hz.

**Figure 21.**
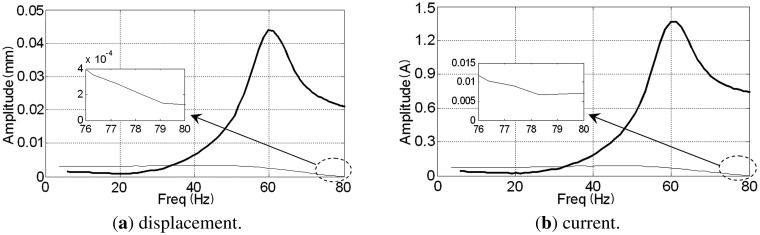
Amplitude of synchronous displacement and current of AX-channel from 80 Hz to 0 before (bold curve) and after (thin curve) balancing at 80 Hz.

The rotor spins at frequency 80 Hz under the PD controller, and a comparison of the rotor displacements before and after balancing is given in Figure 20. After balancing, we find that the displacements at the *B*-end are nearly zero and the fluctuations at the *A*-end are the high-frequency rough target-detection error rather than the synchronous signal [29]. The amplitudes of the synchronous displacement and current of *AX*-channel are extracted using the method discussed in Section 5.1 when the rotor speeds down from 80 Hz to zero (see Figure 21). The peaks of the curves before balancing in the figure are located at the rigid critical speed of the MLR system. The rotor displacement decreases from 21 μm to 0.14 μm and coil current decreases from 740 mA to 7 mA at the balancing frequency (80 Hz).

**Figure 22.**
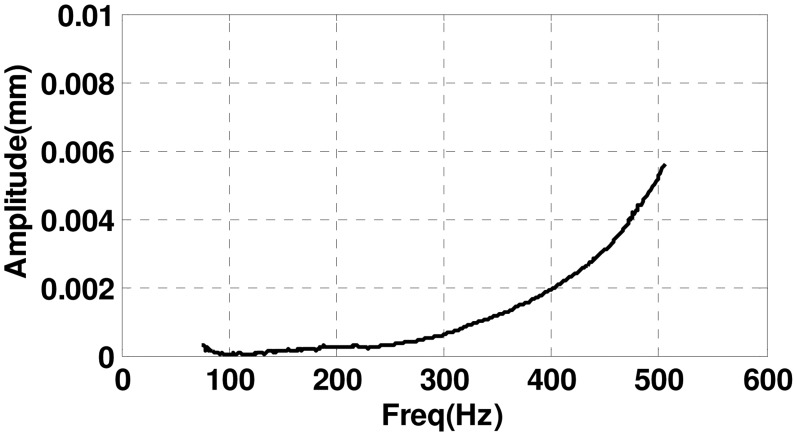
Amplitude of rotor's synchronous displacement after field balancing with the rotation speed decreasing from 500 Hz to 0.

Figure 22 is the amplitude of rotor's synchronous displacement from 500 Hz to 0 after field balancing. We can see that rotor's displacement is smaller than 0.3 μm when the speed is below 250 Hz. Rotor displacement improves rapidly after 300 Hz, and the flexible unbalance begins to affect rotor's displacement. When the rotor spins above 400 Hz, flexible unbalance plays a key role in rotor displacement, and flexible balancing must be carried out.

## 7. Conclusions

A new method of field dynamic balancing for an MLR has been proposed that requires no trial weights or rotor-bearing model, but which can make balancing simultaneously highly efficient and highly accurate. First, the MLR dynamics including static unbalance and dynamic unbalance was modeled. According to the models, we found that making the rotor's rotation axis align with the geometric axis would bring two benefits. One was that the unbalanced centrifugal force/torque equaled the synchronous magnetic force/torque generated by the control current; the other is that the magnetic force was proportional to the control current, assisting in making field balancing highly accurate. Second, using the UCC method with a GPF enabled the MLR to spin around its geometric axis. Finally, the correction masses was calculated using control current rather than rotor displacement, which required only a single startup, and particularly without the need for influence coefficients. The experimental results showed that this novel balancing method increased the balance grade by two orders of magnitude based on off-line balancing, achieving 0.024 g mm kg^−1^. Though the proposed balancing method performs well in the experiment, it still has many limitations as follows:

(1) It is effective only for rigid rotors;
(2) The off-line balancing is indispensable. The proposed method requires that the magnetic force should be able to counterbalance the unbalance centrifugal force, so it is not effective for rotors that have large unbalances;
(3) The rotor's mass distribution should change little in operation. Otherwise, an active vibration control method should be used in conjunction with the propose method.

## Figures and Tables

**Table 1.** Design parameters of the 4 kW MLM.

**Symbol**	**Name**	**Value**	**Unit**
*L_r_*	Length of the rotor	554	mm
*M*	Mass of the rotor	6.85	kg
*P*	Power	4	kW
*f*_bending_	The first bending frequency	650	Hz
*J_r_*	The transverse moment of inertia	0.1147	kg m^2^
*J_z_*	The polar moment of inertia	0.002529	kg m^2^
*I*_0_	Bias current	1.2	A
*k_i_*	Current stiffness	43	N A^−1^
*k_s_*	Negative position stiffness	−0.21 × 10^6^	N m^−1^
*S*_0_	Radial protective clearance	0.2	mm
*L*_1_	The distance from balancing plane-A to AMB-*A*	166	mm
*L*_2_	The distance of the two AMBs	193	mm
*L*_3_	The distance from AMB-*B* to balancing plane-*B*	168.5	mm
*r*_1_	The correction radius of balancing plane-*A*	20	mm
*r*_2_	The correction radius of balancing plane-*B*	15	mm

**Table 2.** The measured results of the current stiffness.

**Channel**	**Value**	**Unit**
AX	33.45	N A^−1^
AY	33.99	N A^−1^
BX	37.77	N A^−1^
BY	36.06	N A^−1^
